# Two cases of uterine leiomyosarcoma misdiagnosed as intravenous leiomyomatosis before surgery: A case report

**DOI:** 10.1097/MD.0000000000043961

**Published:** 2025-09-19

**Authors:** Yixuan Yang, Guotao Ma, Zhichao Lai, Xiao Ma, Dongyan Cao, Kang Li, Jiang Shao, Bao Liu

**Affiliations:** a Eight-year Medical Doctor Program, Chinese Academy of Medical Sciences & Peking Union Medical College, Beijing, China; b Department of Vascular Surgery, Peking Union Medical College Hospital, Beijing, China; c Department of Cardiac Surgery, Peking Union Medical College Hospital, Beijing, China; d National Clinical Research Center for Obstetric & Gynecologic Diseases, Department of Obstetrics and Gynecology, Peking Union Medical College Hospital, Beijing, China.

**Keywords:** case report, intraoperative pathology, intravenous leiomyomatosis, misdiagnosis, uterine leiomyosarcoma

## Abstract

**Rationale:**

Intravenous leiomyomatosis (IVL) and uterine leiomyosarcoma (ULMS) share overlapping imaging features, leading to frequent preoperative misdiagnosis. Accurate differentiation is critical for appropriate surgical management.

**Patient concerns:**

Case 1 is a 48-year-old woman with intermittent vaginal bleeding. Case 2 is a 52-year-old woman with exertional dyspnea for 8 months.

**Diagnoses:**

Preoperative computed tomography venography suggested IVL in both cases. Final pathology showed case 1 for low-grade endometrial stromal sarcoma with vascular invasion and case 2 for ULMS.

**Interventions:**

Case 1 under hysterectomy, bilateral salpingoo-opherectomy, ureteral replantation, tumor resection. Case 2 under resection of uterus, adnexa, right kidney, and intravascular tumor.

**Outcomes:**

Both cases showed a fine postoperative condition with no severe adverse reactions.

**Lessons:**

Intraoperative pathology is essential for distinguishing ULMS from IVL when imaging is inconclusive.

## 1. Introduction

Intravenous leiomyomatosis (IVL) is a disease that rarely occurs in patients with leiomyoma. Its main characteristic is that histologically benign smooth muscle hyperplasia spreads to the uterus, pelvic vein, and inferior vena cava (IVC) in the form of worms, sometimes reaching as far as the heart.^[1]^ However, owing to its nonspecific clinical features and unusual presentation, IVL can be challenging to diagnose and is frequently misdiagnosed as other venous tumors, such as leiomyosarcoma.^[2]^ Uterine leiomyosarcoma (ULMS) is a malignant smooth muscle tumor that accounts for ~3% to 7% of all uterine malignancies and can present with nonspecific symptoms such as abdominal pain, vaginal bleeding, and a pelvic mass.^[3]^ They have a fleshy appearance and may contain areas of necrosis, bleeding, or cyst formation.^[4]^ It is important to correctly diagnose ULMS as early as possible because misdiagnosis or delayed diagnosis of ULMS can lead to unfavorable outcomes, including advanced disease stage and decreased survival rates.

However, ULMS is clinically indistinguishable from IVL. In general, imaging modalities are important in the diagnosis of IVL. However, imaging has difficulty differentiating between features of IVL and ULMS because both show that the local mass appears in the uterus.

Our hospital receives a very large proportion of all IVL patients.^[5,6]^ In this case study, we present 2 patients who were diagnosed with IVL before surgery, but their intraoperative characteristics showed that they were actually ULMS. We noted that it is difficult to distinguish the 2 during preoperative examination, so it is important to pay attention to the results of macroscopic examination, frozen pathology during surgery, and immunohistochemical analysis. Additionally, we noted several other measures that can possibly distinguish between the 2 diseases to correctly and promptly diagnose ULMS according to a literature review. Both patients mentioned in this case report consented to the publication of this manuscript.

## 2. Case 1 presentation

A 48-year-old woman with a history of chronic illness presented to our hospital with intermittent vaginal bleeding for the past few months. She had been receiving treatment for uterine leiomyoma for more than a year. An angioleiomyoma was discovered 2 months prior to admission. Her medical history was notable for a previous cesarean section in 2002. Physical examination revealed a 10-cm horizontal surgical scar in the lower abdomen, abdominal distension, and an enlarged uterus palpable 2 fingerbreadths below the umbilicus.

**Figure 1.**
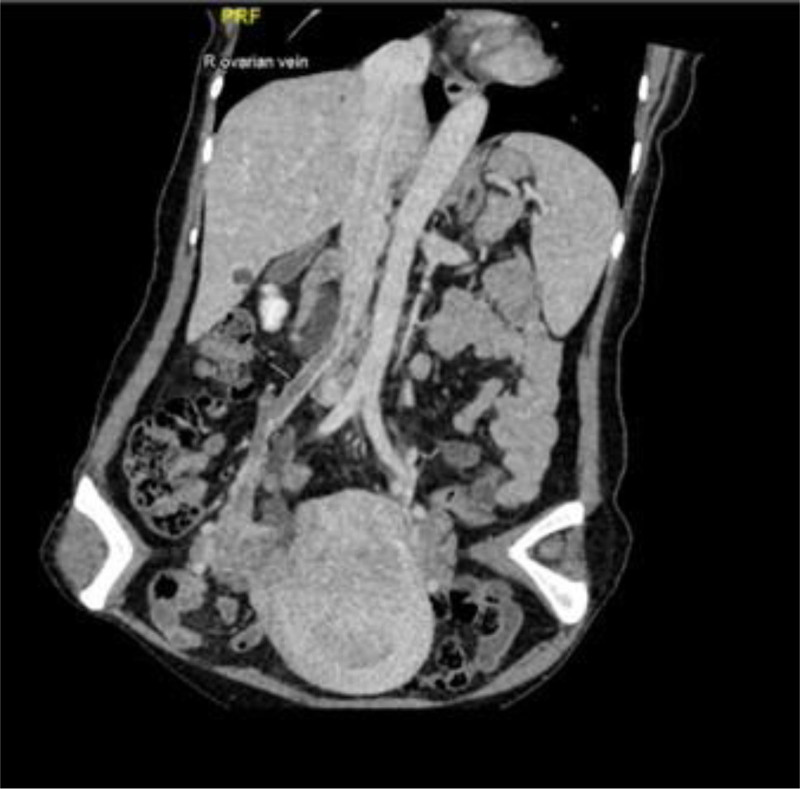
Lower limb deep venous CTV of case 1. CTV showed a mass located in pelvis, extending from the right internal iliac vein to the right atrium, supporting IVL. CTV = computed tomography venography, IVL = intravenous leiomyomatosis.

Further investigations, including our hospital’s IVC and lower limb deep venous computed tomography venography (CTV), revealed filling defects in the IVC, right common iliac vein, right internal iliac vein, and right gonadal vein. The distal end of the right ureter was also surrounded by the lesion. CTV imaging revealed a mass originating from the right internal iliac vein, with a diameter of ~1 cm, that extended upward to the right atrium (Fig. 1). The findings of the present study were consistent with the possibility of IVL.

Considering the patient’s medical history, physical examination, and consultation with multiple specialties, a diagnosis of IVL was established.

**Figure 2.**
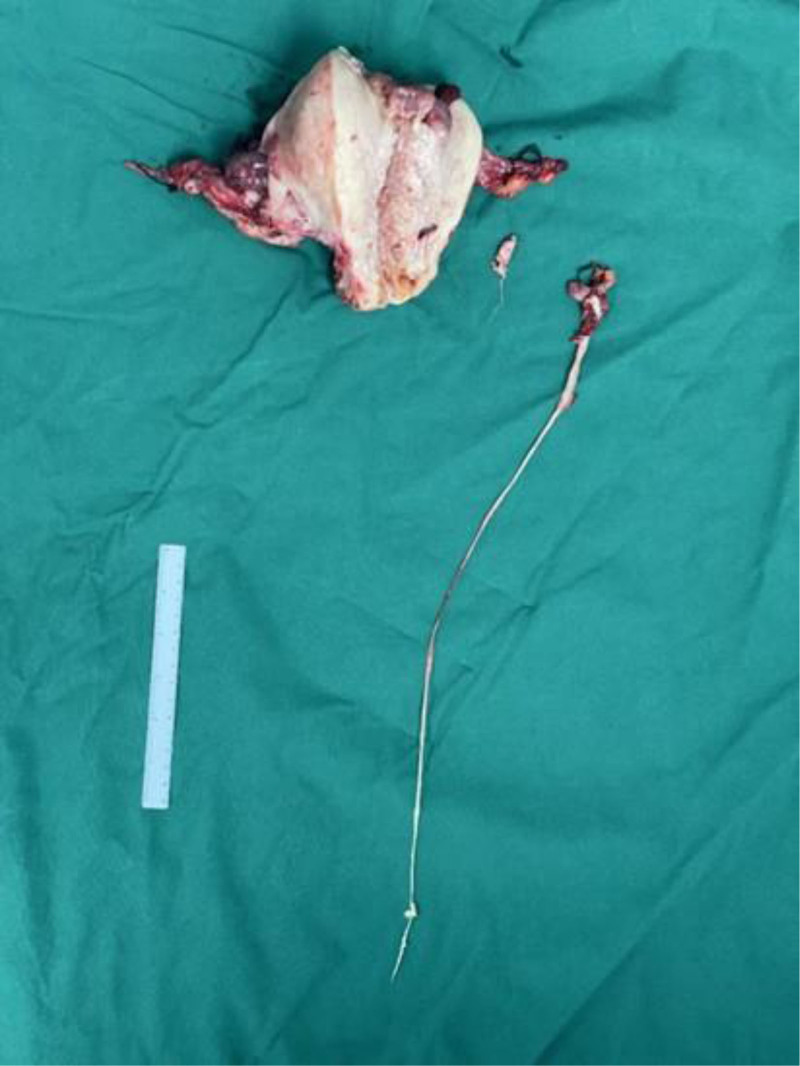
Case 1 intraoperative resection of uterus and its appendages and intravenous tumors.

The patient underwent laparotomy, hysterectomy plus bilateral salpingoo-opherectomy ureteral bladder replantation, and resection of the IVL during surgery. Intraoperative pathology revealed a low-grade endometrial stromal sarcoma in the uterus and both adnexae. The tumor extensively involved the uterine wall, with numerous intravascular tumor thrombi observed. The tumor also infiltrated the cervical tissue, with a margin of <2 mm from the outer os. The tumor additionally involved the right adnexa and surrounding vasculature. The endometrium was in the proliferative phase. A right genital vein tumor and right internal iliac vein tumor were also noted, which was consistent with the diagnosis of low-grade endometrial stromal sarcoma (Fig. 2). The immunohistochemical results in Table 1. Intraoperative pathology led to the final diagnosis of ULMS. The operation image is shown below. Patient went well after this surgery.

**Table 1:** Immunohistochemical staining results for case 1.

Marker	Result
CD10	Positive (+)
Caldesmon	Negative (−)
Desmin	Negative (−)
ER	Positive (+)
Ki-67	20%
SMA	Negative (−)
PR	Partially +
Bcl-2	Positive (+)
Cyclin D1	Negative (−)
VEGF	Negative (−)

This table summarizes the immunohistochemical profile of the tumor for case 1.

ER = estrogen receptor, PR = progesterone receptor, SMA = smooth muscle actin, VEGF = vascular endothelial growth factor.

*Outcome*: The patient recovered well postoperatively, with no recurrence observed at 6-month follow-up.

## 3. Case 2 presentation

A 52-year-old woman presented to our hospital with shortness of breath after exercise for 8 months, which improved after rest. An ultrasonic cardiogram revealed a “right heart mass, continuous abnormal echo of the inferior vena cava.” A complete enhanced computed tomography (CT) scan of the abdomen revealed “consideration of a malignant tumor originating from the right ovarian vein, invading the left renal vein, IVC, right atrium, bilateral iliac veins, and right uterine vein.”

**Figure 3.**
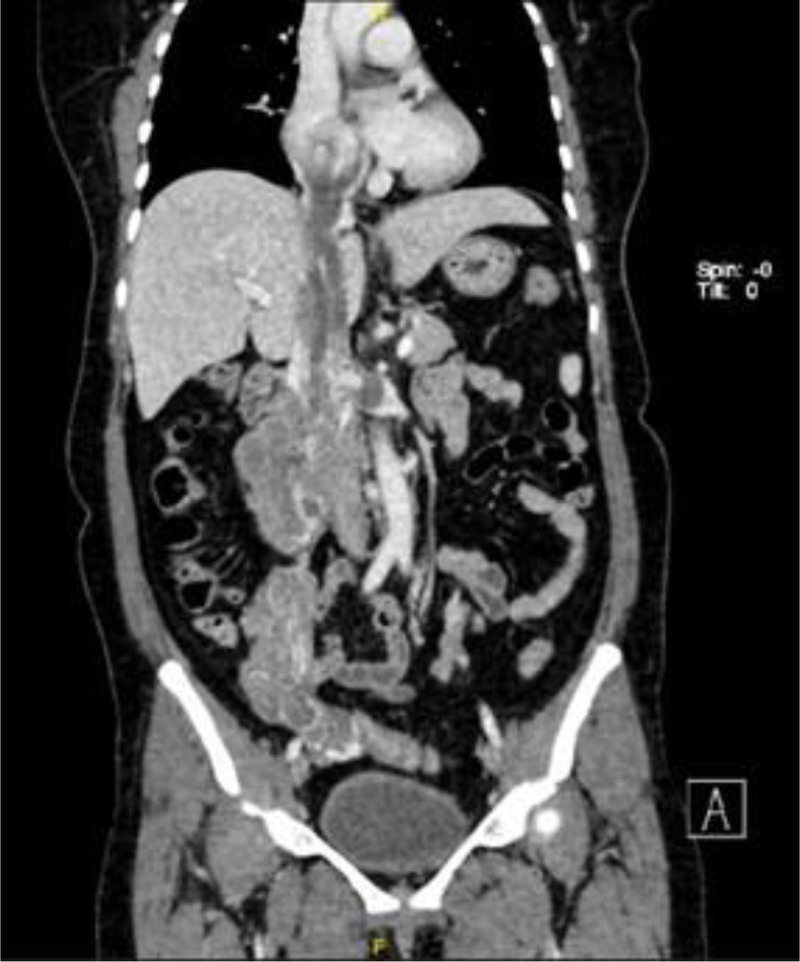
Lower limb deep venous CTV of case 2. CTV showed an uneven mass stretching from the right ovarian veins to the right atrium, supporting IVL. CTV = computed tomography venography, IVL = intravenous leiomyomatosis.

A deep venous CTV of the lower limb revealed a mass of equal density near the right renal hilum and pelvic cavity, with a maximum cross-section of 69.6 mm × 59.8 mm (Fig. 3). The lesion surrounded and invaded the lower vena cava and the left renal vein near the segment and extended upward along the vena cava system to the right ventricle and right atrium and downward to the uterus and bilateral reproductive veins. The right renal artery was surrounded by the lesion, the lumen did not show significant stenosis, and the right renal vein showed a change in pressure. The right ureter was indistinct, and the calyces of the above renal pelvis were dilated and hydronephrotic. All the findings above supported the diagnosis of IVL.

**Figure 4.**
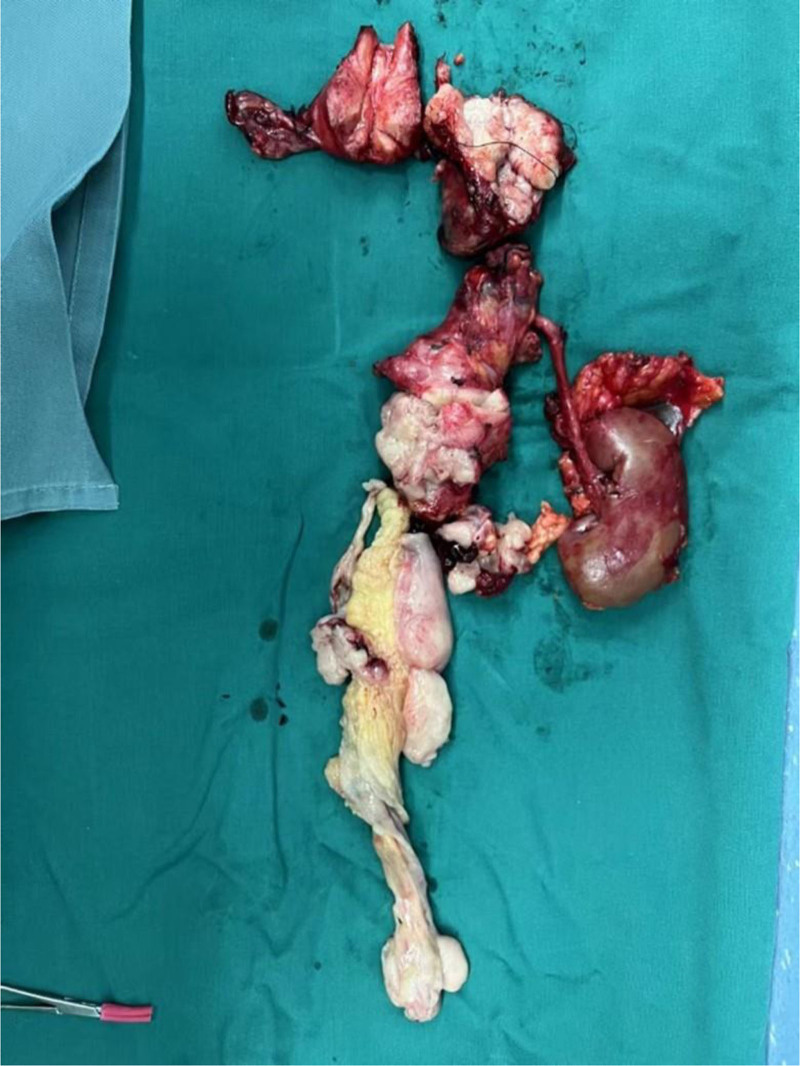
Case 2 intraoperative resection of uterus and its appendages, right kidney, and intravenous tumor.

Considering the patient’s sex, age, and auxiliary examination results, the growth characteristics, which originate from the reproductive veins and grow upward along the venous system to the IVC and then to the right heart, are consistent with the characteristics of IVL. The patient underwent surgery accordingly. During surgery, the size of the tumor was found not to meet the expected size of a typical IVL mass (Fig. 4). Additionally, separating the tumor was more difficult than expected because severe adhesion occurred between the tumor and the vascular wall. The immunohistochemistry results in Table 2. On the basis of the intraoperative pathology and immunohistochemistry results, the patient was finally diagnosed with ULMS. Patient went well after this surgery.

**Table 2:** Immunohistochemical staining results for case 2.

Marker	Result
Caldesmon	Positive (+)
Desmin	Positive (+)
Ki-67	80%
SMA	Positive (+)
CD34	Weak positive
S-100	Negative (−)
AE1/AE3	Positive (+)/negative (−)
Calponin	Positive (+)
CD68	Scattered positive
FH	Positive (+)

This table summarizes the immunohistochemical staining profile of the tumor for case 2. Ki-67 is reported as a proliferation index (%). Weak positive indicates focal or low-intensity staining. Scattered positive refers to patchy or nonuniform immunoreactivity.

AE1/AE3 = pan-cytokeratin (epithelial marker), FH = fumarate hydratase, SMA = smooth muscle actin.

*Outcome*: No complications reported during 8-month follow-up.

## 4. Discussion

IVL histologically shows the characteristics of typical uterine leiomyomas and is closely related to sex hormones. The IVL is actually a type of benign tumor when it is observed at the gross and microscopic levels. However, it can have aggressive invasion, such as malignant tumors extending outside the uterus along the vasculature and spreading to the iliac vein and IVC.^[7]^ It usually has no stalks, and it can reach the right heart without adhering to the wall,^[1]^ which is a special characteristic that distinguishes it from other diseases. The diagnosis of IVL is usually based on imaging examinations, such as CT, color Doppler ultrasound, and magnetic resonance imaging (MRI). A typical acoustic image shows multiple hypoechoic nodules between the muscle wall fusing with each other and the arborizing or strip-shaped blood supply at the fibroid site.^[7]^ When the lesion involved the iliac, ovarian, and IVC, there was marked luminal expansion during the pressurized probe, which was visible as uneven rods of moderate echogenic quality and no deformed veins.^[8]^ The right-sided cardiac tumor exhibited homogeneous echogenicity and was not attached to the right wall or vein wall of the heart.^[7]^ If imaging reveals uterine fibroids or deformation and protrusion into the vascular system, it should reveal the possibility of IVL. For its treatment, most cases need to be treated by surgical resection of intravascular masses.^[9–11]^

However, interestingly, in the 2 cases we presented above, even though the physical and imaging characteristics revealed typical IVL, the results of the intraoperative pathology were completely different. Finally, the conclusion of the intraoperative pathology was actually true, which means that we failed to correctly distinguish UMSL from IVL before surgery, even if conventional imaging methods were used and analyzed.

The UMSL is a type of malignant tumor that has characteristics such as coagulative necrosis of cells and tissue adhesion. In addition, it is rarely possible to reach the heart site from the uterus all the way along the blood vessels, which contributes to the misdiagnosis in these 2 cases. Therefore, when patients present with the possibility of IVL, differential diagnosis with UMSL must be considered. There are several measures to distinguish if it is UMSL. When UMSL is suspected on the basis of ultrasound, MRI with gadolinium as a contrast agent may be helpful in assessing the likelihood of malignant disease, and it has a high negative predictive value when there are typical findings of uterine fibroids.^[12]^ Another important diagnosis for USML is the absence of calcification.^[13]^ Some data have also shown that unclear boundaries are also consistent with the characteristics of the USML.^[14]^ Diffusion-weighted MRI also seems to be able to distinguish common and degenerative leiomyomas from USMLs and cellular leiomyomas.^[15]^ Finally, bleeding in the lesion seems to indicate USML.^[16]^ On the other hand, until now, neither CT nor FDG-PET/CT has been able to reliably differentiate uterine leiomyoma from uterine sarcoma.^[17,18]^

The most important step in diagnosing USML is intraoperative pathology. Typically, intraoperative pathology is not necessary in IVL surgery. However, in both cases mentioned above, several abnormal characteristics were observed during surgery, including heavy partial adhesion and a pelvic mass that is not as large as usual in IVL. These findings indicate specific characteristics of malignancy. Eventually, the result of the intraoperative pathology revealed the USML. USML tumors typically invade through the myometrium, with occasional cases presenting as polypoid growth in the endometrial cavity; these tumors generally have a soft and bulging cut surface that displays scattered areas of hemorrhage and necrosis.^[19]^ The marked histological features include spindle cells with blunt end nuclei, atypical forms of mitotic activity (>10 mitoses per 10 high-power fields), nuclear pleomorphism, excessive cells with fascicular growth patterns, coagulative necrosis, and infiltration of the surrounding myometrium.^[20]^ However, these features can also be observed in many leiomyomas.^[21]^ Coagulative necrosis, not infarct or hyaline necrosis (which can occur in benign as well as malignant lesions), can act as a specific feature of USML; other cytological features that should exist are the phenotypic characteristics of smooth muscle cells, including fibrous and eosinophilic cytoplasm, as well as blunt terminal nuclei.^[20]^ Women with advanced USML have a poor prognosis, although most of them present with diseases limited to local areas of the uterus or pelvis; a considerable portion (30%–35%) also experience metastasis.^[22]^

For the treatment strategy, USML is also different from IVL and needs more measures. Compared with IVL, USML usually requires the excision of more areas during surgery and has a poor prognosis. When patients are treated with surgery, IVL requires only resection of the uterus and double appendages, whereas the ULMS requires surgical steps such as lymph node dissection, which is included in the treatment of the malignancy. For the early stage of USML, adjuvant therapy, such as radiation and chemotherapy, lacks proven benefit.^[19]^ However, chemotherapy effectively increases the survival rate of women with advanced or recurrent USML.^[23]^ If misdiagnosis occurs before surgery and intraoperative pathology is ignored, treatment will be significantly delayed, and a poor prognosis can be predicted.

However, this study is limited by its small case series (n = 2), which precludes statistical analysis and comparison with control groups. Findings should be interpreted with caution pending validation in larger cohorts.

In conclusion, there are several possible methods for differentially diagnosing IVL and USML. In fact, IVL is a rare disease, whereas USML is even rarer. It is difficult to diagnose these rare diseases, and misdiagnosis of USML is very unfavorable for the treatment and survival of patients. Given that reliable means of diagnosis are currently insufficient, we need to pay more attention to the results of intraoperative pathology, especially in the differential diagnosis of these 2 diseases, preventing preoperative hamstring diagnosis.

## Author contributions

**Conceptualization:** Yixuan Yang, Xiao Ma, Dongyan Cao, Bao Liu.

**Investigation:** Xiao Ma.

**Methodology:** Guotao Ma.

**Resources:** Yixuan Yang, Guotao Ma, Dongyan Cao, Kang Li, Jiang Shao, Bao Liu.

**Supervision:** Zhichao Lai, Jiang Shao.

**Writing – original draft:** Yixuan Yang.

**Writing – review & editing:** Yixuan Yang, Zhichao Lai, Kang Li, Bao Liu.
